# Supplementary material for: Axonal spheroids are regulated by Schwann cells after peripheral nerve injury
Source: bioRxiv. 2024 Nov 8:2024.11.08.622649. Preprint. [Version 1] doi: 10.1101/2024.11.08.622649 (PMC11581001; doi:10.1101/2024.11.08.622649)
Supplement: Supplement 9 [file media-9.docx]

Spheroid Quantities SC Ablation Poisson

Marieke Jones and Sarah Hunter-Chang

2024-10-21

## Purpose

Purpose: fit a Poisson mixed effects model to individual zebrafish injury spheroid quantities over time with (Ronidazole, RDZ, treated) and without (Vehicle control, VC, treated) Schwann cell ablation

library(tidyverse)
library(ggeffects)
library(emmeans)
library(lme4)
library(splines)

## Import the data set

Data should be in long form with a column for time, zebrafish ID, RDZ status, and number of spheroids at each time

mydata <- read_csv("Spheroid_quantities_long.csv")

Remove empty columns

mydata <- mydata[,1:5]

Generate a count of how many observations per fish and per treatment

mydata %>%
 count(RDZ, Fish)

## # A tibble: 20 × 3
## RDZ Fish n
## <chr> <chr> <int>
## 1 RDZ G 60
## 2 RDZ H 60
## 3 RDZ I 60
## 4 RDZ J 60
## 5 RDZ K 60
## 6 RDZ L 60
## 7 RDZ M 60
## 8 RDZ N 60
## 9 RDZ O 60
## 10 RDZ P 60
## 11 RDZ Q 60
## 12 RDZ R 60
## 13 RDZ S 60
## 14 RDZ T 60
## 15 VC A 60
## 16 VC B 60
## 17 VC C 60
## 18 VC D 60
## 19 VC E 60
## 20 VC F 60

## Make Time into a minutes variable instead of clock time

First make Time a proper Time variable

mydata <- mydata %>%
 drop_na() %>%
 mutate(Time = hms(Time))

mydata

## # A tibble: 1,200 × 5
## Time Fish RDZ n_spheroids rel_spheroids
## <Period> <chr> <chr> <dbl> <dbl>
## 1 5M 0S A VC 0 0
## 2 10M 0S A VC 0 0
## 3 15M 0S A VC 0 0
## 4 20M 0S A VC 0 0
## 5 25M 0S A VC 4 11.0
## 6 30M 0S A VC 7 19.3
## 7 35M 0S A VC 14 38.5
## 8 40M 0S A VC 15 41.3
## 9 45M 0S A VC 16 44.0
## 10 50M 0S A VC 19 52.3
## # ℹ 1,190 more rows

Then get the number of minutes past nerve ablation

mydata <- mydata %>%
 mutate(Min = as.duration(Time) / dminutes(1))

mydata %>%
 print(n = 30)

## # A tibble: 1,200 × 6
## Time Fish RDZ n_spheroids rel_spheroids Min
## <Period> <chr> <chr> <dbl> <dbl> <dbl>
## 1 5M 0S A VC 0 0 5
## 2 10M 0S A VC 0 0 10
## 3 15M 0S A VC 0 0 15
## 4 20M 0S A VC 0 0 20
## 5 25M 0S A VC 4 11.0 25
## 6 30M 0S A VC 7 19.3 30
## 7 35M 0S A VC 14 38.5 35
## 8 40M 0S A VC 15 41.3 40
## 9 45M 0S A VC 16 44.0 45
## 10 50M 0S A VC 19 52.3 50
## 11 55M 0S A VC 12 33.0 55
## 12 1H 0M 0S A VC 17 46.8 60
## 13 1H 5M 0S A VC 23 63.3 65
## 14 1H 10M 0S A VC 27 74.3 70
## 15 1H 15M 0S A VC 31 85.3 75
## 16 1H 20M 0S A VC 28 77.1 80
## 17 1H 25M 0S A VC 26 71.6 85
## 18 1H 30M 0S A VC 26 71.6 90
## 19 1H 35M 0S A VC 26 71.6 95
## 20 1H 40M 0S A VC 24 66.1 100
## 21 1H 45M 0S A VC 26 71.6 105
## 22 1H 50M 0S A VC 23 63.3 110
## 23 1H 55M 0S A VC 27 74.3 115
## 24 2H 0M 0S A VC 33 90.8 120
## 25 2H 5M 0S A VC 30 82.6 125
## 26 2H 10M 0S A VC 35 96.3 130
## 27 2H 15M 0S A VC 31 85.3 135
## 28 2H 20M 0S A VC 31 85.3 140
## 29 2H 25M 0S A VC 29 79.8 145
## 30 2H 30M 0S A VC 26 71.6 150
## # ℹ 1,170 more rows

Yes, this worked

Update 2024-10-18. SHC says “data set should start at 15 minutes, not 5. I was looking over things again and remembered that some of my time lapses started >10 minutes after injury. I’d automatically filled every empty time point with a 0 because the spheroid detection software just leaves time points in which it detects 0 spheroids blank, even if the time lapse included that time point. So now some of the spheroid quantities from <15 minutes were actually unknown, but erroneously entered as 0”

Subset for 15+ minutes

mydata <- mydata %>%
 filter(Min >= 15)

Now regenerate the count of how many observations per fish and per treatment

mydata %>%
 count(RDZ, Fish)

## # A tibble: 20 × 3
## RDZ Fish n
## <chr> <chr> <int>
## 1 RDZ G 58
## 2 RDZ H 58
## 3 RDZ I 58
## 4 RDZ J 58
## 5 RDZ K 58
## 6 RDZ L 58
## 7 RDZ M 58
## 8 RDZ N 58
## 9 RDZ O 58
## 10 RDZ P 58
## 11 RDZ Q 58
## 12 RDZ R 58
## 13 RDZ S 58
## 14 RDZ T 58
## 15 VC A 58
## 16 VC B 58
## 17 VC C 58
## 18 VC D 58
## 19 VC E 58
## 20 VC F 58

## Plots

Make a spaghetti plot showing **number of spheroids** over time for each individual fish

mydata %>%
 ggplot(aes(Min, n_spheroids)) +
 geom_line(aes(group = Fish)) +
 facet_wrap(vars(RDZ))


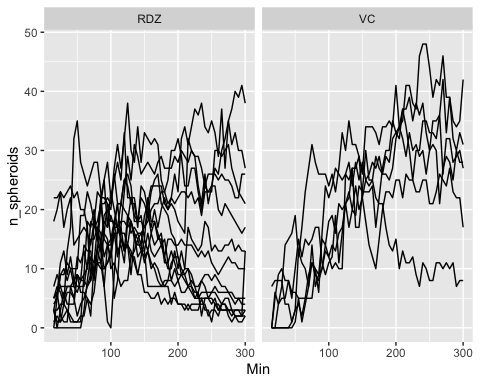


# see one fish
mydata %>%
 filter(Fish == "A") %>%
 ggplot(aes(Min, n_spheroids)) +
 geom_line(aes(group = Fish)) +
 facet_wrap(vars(RDZ))


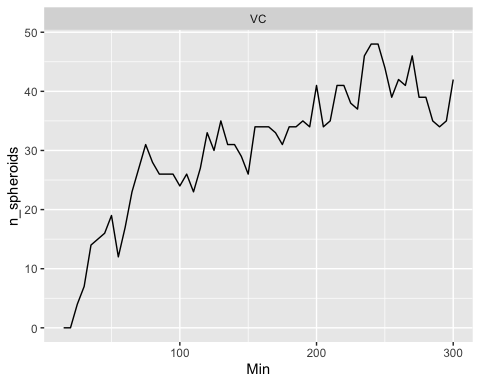


Regenerate the plot from Prism

Calculate the average n_spheroid at each time point for each drug treatment group. Put VC first.

mydataSUM <- mydata %>%
 group_by(RDZ, Min) %>%
 summarize(mean_spheroids = mean(n_spheroids),
 sem_spheroids = sd(n_spheroids) / sqrt(n()),
 lower = mean_spheroids - sem_spheroids,
 upper = mean_spheroids + sem_spheroids,
 .groups = "drop") %>%
 mutate(RDZ = factor(RDZ, levels = c("VC", "RDZ")))

mydataSUM

## # A tibble: 116 × 6
## RDZ Min mean_spheroids sem_spheroids lower upper
## <fct> <dbl> <dbl> <dbl> <dbl> <dbl>
## 1 RDZ 15 5.64 1.72 3.92 7.36
## 2 RDZ 20 6.57 1.79 4.78 8.36
## 3 RDZ 25 7.57 1.97 5.61 9.54
## 4 RDZ 30 7.43 1.68 5.75 9.11
## 5 RDZ 35 8.21 1.80 6.42 10.0
## 6 RDZ 40 9.21 1.97 7.24 11.2
## 7 RDZ 45 8.86 2.34 6.51 11.2
## 8 RDZ 50 9.93 2.54 7.39 12.5
## 9 RDZ 55 9.86 2.17 7.68 12.0
## 10 RDZ 60 11.3 1.90 9.39 13.2
## # ℹ 106 more rows

Now plot

mydataSUM %>%
 ggplot(aes(Min, mean_spheroids, color = RDZ, fill = RDZ)) +
 geom_line(linewidth = 2) +
 geom_ribbon(aes(ymax = upper, ymin = lower),
 alpha = .2,
 color = NA)


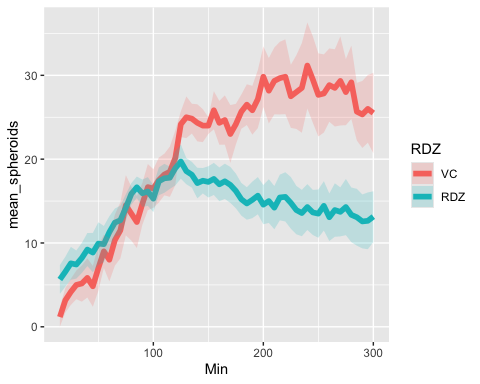


## Fit Poisson mixed effects model

mod1 <- glmer(n_spheroids ~ Min * RDZ + (1|Fish), family = poisson, data = mydata)
summary(mod1, corr = FALSE)

## Generalized linear mixed model fit by maximum likelihood (Laplace
## Approximation) [glmerMod]
## Family: poisson ( log )
## Formula: n_spheroids ~ Min * RDZ + (1 | Fish)
## Data: mydata
##
## AIC BIC logLik deviance df.resid
## 9207.0 9232.3 -4598.5 9197.0 1155
##
## Scaled residuals:
## Min 1Q Median 3Q Max
## -4.8321 -1.2831 0.0761 1.1734 7.8214
##
## Random effects:
## Groups Name Variance Std.Dev.
## Fish (Intercept) 0.1387 0.3725
## Number of obs: 1160, groups: Fish, 20
##
## Fixed effects:
## Estimate Std. Error z value Pr(>|z|)
## (Intercept) 2.4341882 0.1017092 23.933 < 2e-16 ***
## Min 0.0008564 0.0001115 7.682 1.57e-14 ***
## RDZVC -0.2420752 0.1854430 -1.305 0.192
## Min:RDZVC 0.0037607 0.0001843 20.405 < 2e-16 ***
## ---
## Signif. codes: 0 '***' 0.001 '**' 0.01 '*' 0.05 '.' 0.1 ' ' 1
## optimizer (Nelder_Mead) convergence code: 0 (OK)
## Model is nearly unidentifiable: very large eigenvalue
## - Rescale variables?
## Model is nearly unidentifiable: large eigenvalue ratio
## - Rescale variables?

See the model in a plot

plot(ggpredict(mod1, terms = c("Min", "RDZ")))


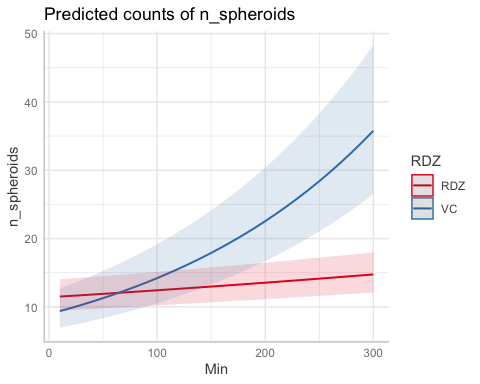


# add the raw data
plot(ggpredict(mod1, terms = c("Min", "RDZ")),
 add.data = T)


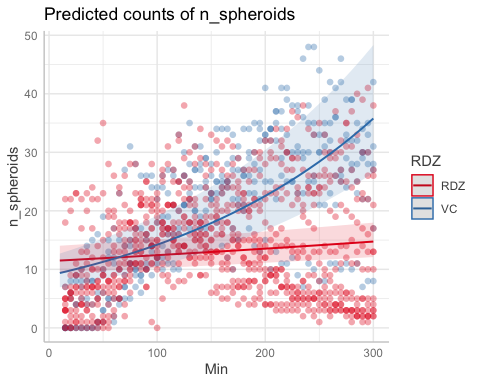


The RDZ do not seem to fit well

See the model for each fish

ggpredict(mod1, terms = c("Min", "RDZ", "Fish"), type = "random") %>%
 as_tibble() %>%
 rename(Min = x,
 RDZ = group,
 Fish = facet) %>%
 ggplot() +
 geom_line(aes(x = Min, y = predicted, color = RDZ)) +
 facet_wrap(~Fish)


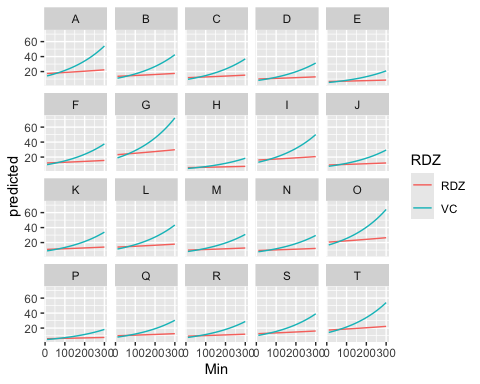


# add the raw data to see the fit
ggpredict(mod1, terms = c("Min", "RDZ", "Fish"), type = "random") %>%
 as_tibble() %>%
 rename(Min = x,
 RDZ = group,
 Fish = facet) %>%
 ggplot() +
 geom_line(aes(x = Min, y = predicted, color = RDZ)) +
 facet_wrap(~Fish) +
 geom_line(data = mydata, aes(Min, n_spheroids, group = Fish, color = RDZ))


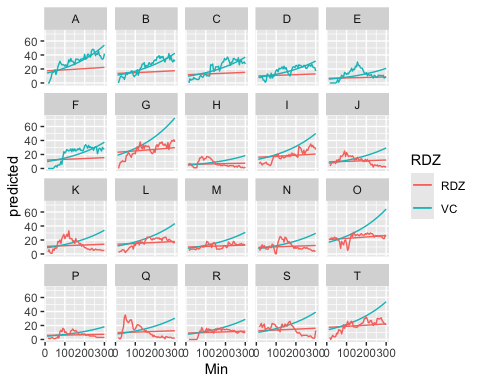


Actually, this model fits better than I thought.

# Poisson mixed effects model with restricted cubic splines

Try with non-linear spline terms in case that complexity is better

mod2 <- glmer(n_spheroids ~ ns(Min, df = 3) + RDZ + (1|Fish),
 family = poisson,
 data = mydata)

See the model

plot(ggpredict(mod2, terms = c("Min [all]", "RDZ")))


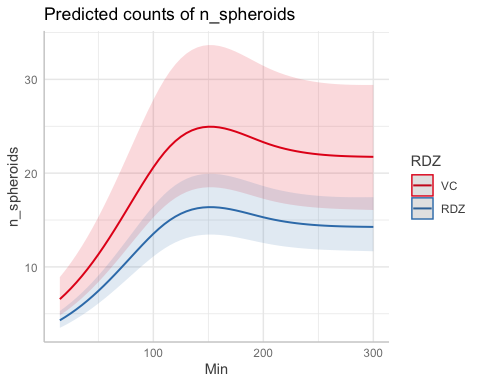


See this model for each fish

ggpredict(mod2, terms = c("Min [all]", "RDZ", "Fish"), type = "random") %>%
 as_tibble() %>%
 rename(Min = x,
 RDZ = group,
 Fish = facet) %>%
 ggplot() +
 geom_line(aes(x = Min, y = predicted, color = RDZ)) +
 facet_wrap(~Fish)


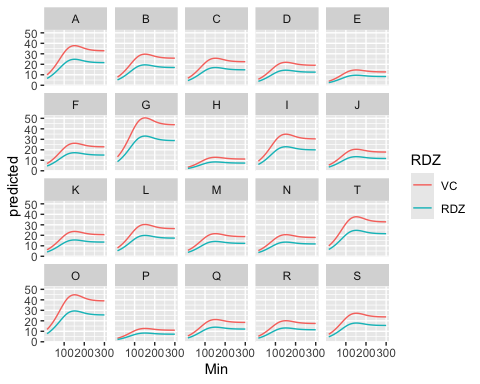


# add the raw data to see the fit
ggpredict(mod2, terms = c("Min [all]", "RDZ", "Fish"), type = "random") %>%
 as_tibble() %>%
 rename(Min = x,
 RDZ = group,
 Fish = facet) %>%
 ggplot() +
 geom_line(aes(x = Min, y = predicted, color = RDZ)) +
 facet_wrap(~Fish) +
 geom_line(data = mydata, aes(Min, n_spheroids, group = Fish, color = RDZ))


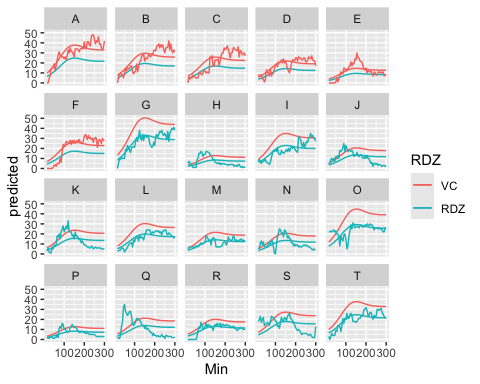


Hmm, not sure this version is better

AIC(mod1, mod2)

## df AIC
## mod1 5 9207.010
## mod2 6 8719.908

According to AIC, mod2 does have a better fit

## Try splines with an interaction term

mod3 <- glmer(n_spheroids ~ ns(Min, df = 3) * RDZ + (1|Fish),
 family = poisson,
 data = mydata)

See the model

plot(ggpredict(mod3, terms = c("Min [all]", "RDZ")))


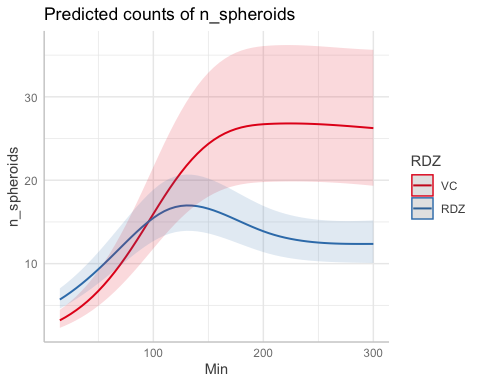


Ahh, this might be better

See this model for each fish

ggpredict(mod3, terms = c("Min [all]", "RDZ", "Fish"), type = "random") %>%
 as_tibble() %>%
 rename(Min = x,
 RDZ = group,
 Fish = facet) %>%
 ggplot() +
 geom_line(aes(x = Min, y = predicted, color = RDZ)) +
 facet_wrap(~Fish)


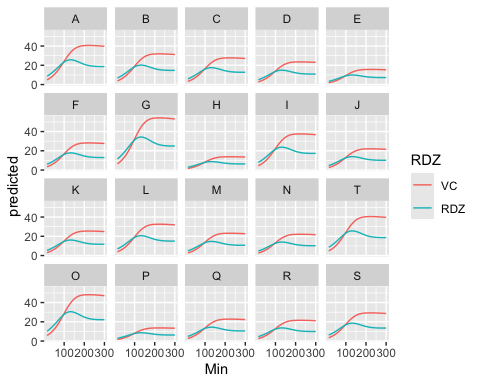


# add the raw data to see the fit
ggpredict(mod3, terms = c("Min [all]", "RDZ", "Fish"), type = "random") %>%
 as_tibble() %>%
 rename(Min = x,
 RDZ = group,
 Fish = facet) %>%
 ggplot() +
 geom_line(aes(x = Min, y = predicted, color = RDZ)) +
 facet_wrap(~Fish) +
 geom_line(data = mydata, aes(Min, n_spheroids, group = Fish, color = RDZ))


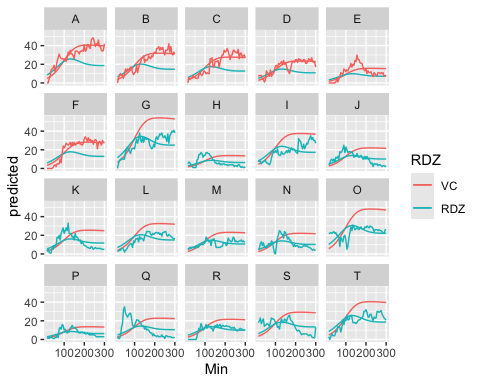


Check out the difference in AIC between these mod2 and mod3

AIC(mod2, mod3)

## df AIC
## mod2 6 8719.908
## mod3 9 8157.726

Mod3 is preferred

See the average raw data plotted with this model

ggpredict(mod3, terms = c("Min [all]", "RDZ")) %>%
 as_tibble() %>%
 rename(Min = x,
 RDZ = group) %>%
 ggplot() +
 geom_line(aes(x = Min, y = predicted, color = RDZ),
 linewidth = 2) +
 geom_line(data = mydataSUM, aes(Min, mean_spheroids, color = RDZ)) +
 geom_ribbon(data = mydataSUM, aes(Min, mean_spheroids, fill = RDZ,
 ymax = upper, ymin = lower),
 alpha = .2,
 color = NA) + # Ribbon with fill by RDZ
 scale_color_manual(values = c("RDZ" = "#FFCC66", "VC" = "#33CCFF")) + # Assign specific colors to RDZ
 scale_fill_manual(values = c("RDZ" = "#FFCC66", "VC" = "#33CCFF")) + # Same colors for ribbon fill
theme_classic() +
 theme(axis.line = element_line(size = 1))


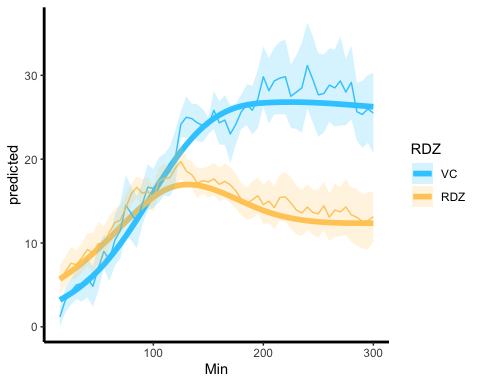


ggsave("plot1.tiff", width = 8, height = 6, dpi = 300)

This model fits these data pretty FREAKIN WELL! I am JAZZED!

See the summary

summary(mod3, corr = FALSE)

## Generalized linear mixed model fit by maximum likelihood (Laplace
## Approximation) [glmerMod]
## Family: poisson ( log )
## Formula: n_spheroids ~ ns(Min, df = 3) * RDZ + (1 | Fish)
## Data: mydata
##
## AIC BIC logLik deviance df.resid
## 8157.7 8203.2 -4069.9 8139.7 1151
##
## Scaled residuals:
## Min 1Q Median 3Q Max
## -4.4111 -1.1783 -0.0869 0.9143 9.6180
##
## Random effects:
## Groups Name Variance Std.Dev.
## Fish (Intercept) 0.1388 0.3725
## Number of obs: 1160, groups: Fish, 20
##
## Fixed effects:
## Estimate Std. Error z value Pr(>|z|)
## (Intercept) 1.739196 0.107505 16.178 <2e-16 ***
## ns(Min, df = 3)1 0.381829 0.039904 9.569 <2e-16 ***
## ns(Min, df = 3)2 1.944269 0.095240 20.414 <2e-16 ***
## ns(Min, df = 3)3 -0.004215 0.033125 -0.127 0.8988
## RDZVC -0.575611 0.200510 -2.871 0.0041 **
## ns(Min, df = 3)1:RDZVC 1.181737 0.069013 17.123 <2e-16 ***
## ns(Min, df = 3)2:RDZVC 1.913358 0.190310 10.054 <2e-16 ***
## ns(Min, df = 3)3:RDZVC 0.947985 0.052985 17.891 <2e-16 ***
## ---
## Signif. codes: 0 '***' 0.001 '**' 0.01 '*' 0.05 '.' 0.1 ' ' 1

Ok, pretty much everything is significant. See the predicted average differences between RDZ and VC at given times

every hour?

60*1:5

## [1] 60 120 180 240 300

See the predicted number of spheroids at given time points

emm <- emmeans(mod3, ~RDZ | Min,
 at = list(Min = c(60, 120, 180, 240, 300)),
 type = "response")

emm

## Min = 60:
## RDZ rate SE df asymp.LCL asymp.UCL
## RDZ 10.6 1.07 Inf 8.68 12.9
## VC 8.2 1.28 Inf 6.04 11.1
##
## Min = 120:
## RDZ rate SE df asymp.LCL asymp.UCL
## RDZ 16.8 1.69 Inf 13.77 20.5
## VC 20.0 3.08 Inf 14.79 27.0
##
## Min = 180:
## RDZ rate SE df asymp.LCL asymp.UCL
## RDZ 15.0 1.51 Inf 12.30 18.3
## VC 26.3 4.03 Inf 19.50 35.5
##
## Min = 240:
## RDZ rate SE df asymp.LCL asymp.UCL
## RDZ 12.7 1.28 Inf 10.39 15.4
## VC 26.8 4.10 Inf 19.83 36.1
##
## Min = 300:
## RDZ rate SE df asymp.LCL asymp.UCL
## RDZ 12.4 1.29 Inf 10.07 15.2
## VC 26.2 4.10 Inf 19.33 35.6
##
## Confidence level used: 0.95
## Intervals are back-transformed from the log scale

Are the drug treatment groups different at each timepoint?

pairs(emm)

## Min = 60:
## contrast ratio SE df null z.ratio p.value
## RDZ / VC 1.291 0.2397 Inf 1 1.376 0.1689
##
## Min = 120:
## contrast ratio SE df null z.ratio p.value
## RDZ / VC 0.839 0.1545 Inf 1 -0.952 0.3411
##
## Min = 180:
## contrast ratio SE df null z.ratio p.value
## RDZ / VC 0.569 0.1043 Inf 1 -3.074 0.0021
##
## Min = 240:
## contrast ratio SE df null z.ratio p.value
## RDZ / VC 0.473 0.0867 Inf 1 -4.086 <.0001
##
## Min = 300:
## contrast ratio SE df null z.ratio p.value
## RDZ / VC 0.471 0.0885 Inf 1 -4.007 0.0001
##
## Tests are performed on the log scale
