# Supplementary material for: Axonal spheroids are regulated by Schwann cells after peripheral nerve injury
Source: bioRxiv. 2024 Nov 8:2024.11.08.622649. Preprint. [Version 1] doi: 10.1101/2024.11.08.622649 (PMC11581001; doi:10.1101/2024.11.08.622649)
Supplement: Supplement 10 [file media-10.pdf]

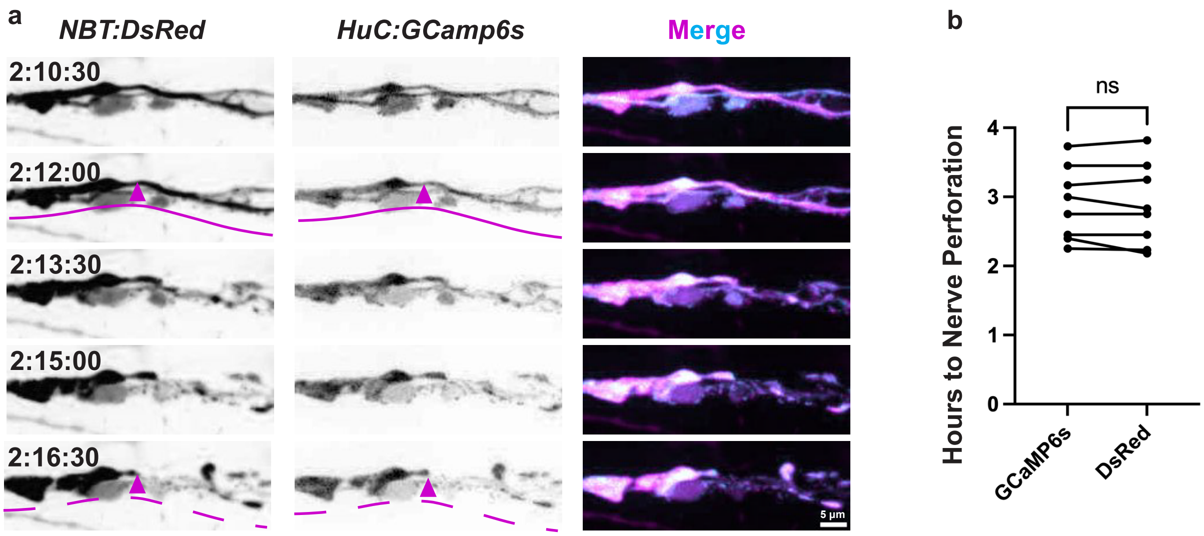

Supplementary Figure 1: GCaMP6s is a valid marker of nerve perforation. a) Intact (solid lines) and perforated (dashed lines) axons (arrowheads) detected by *NBT:DsRed* (left column) and by *HuC:GCaMP6s* (middle column) do not significantly differ in time to nerve perforation (b, N=7 larvae, $p=0.6250$, two-tailed Wilcoxon matched-pairs signed rank test).
