# Supplementary material for: Axonal spheroids are regulated by Schwann cells after peripheral nerve injury
Source: bioRxiv. 2024 Nov 8:2024.11.08.622649. Preprint. [Version 1] doi: 10.1101/2024.11.08.622649 (PMC11581001; doi:10.1101/2024.11.08.622649)
Supplement: Supplement 12 [file media-12.pdf]

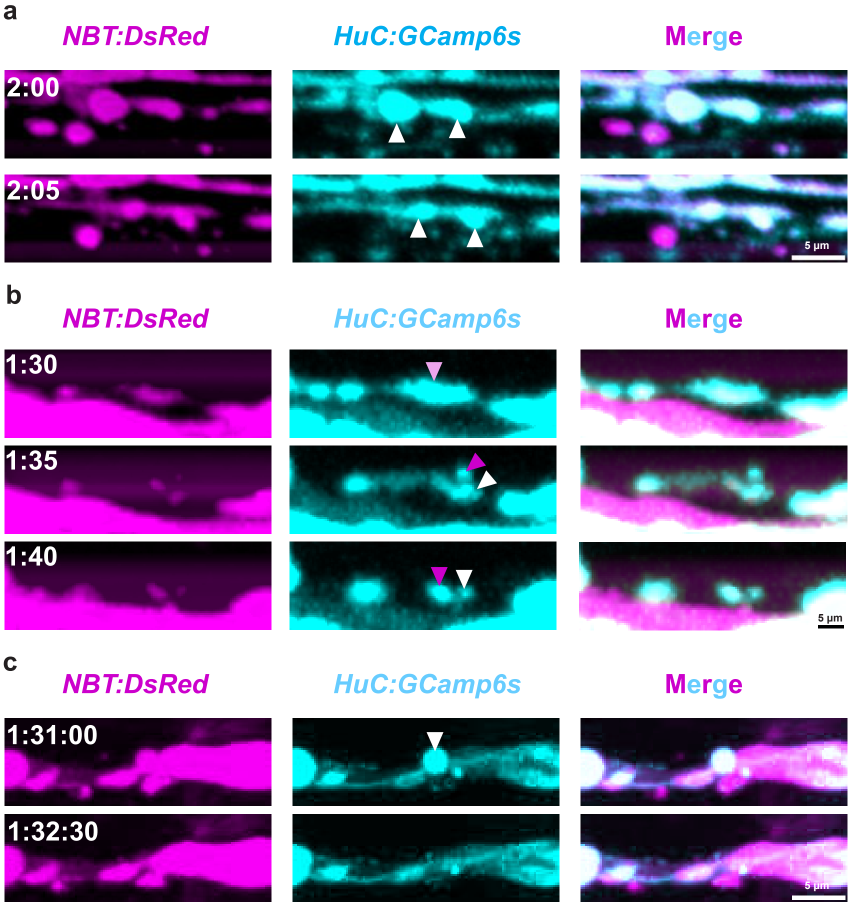

Supplementary Figure 3: Spheroid fates are visible with both *HuC:GCaMP6s* and *NBT:DsRed* labeling. a) Spheroids shrinking (white arrowsheads) in both the neuronal calcium (*HuC:GCaMP6s*) and cytosol (*NBT:DsRed*) channels (N=6 larvae). b) A spheroid (pink arrowhead) fragments (magenta and white arrowheads) visibly with both neuronal calcium (*HuC:GCaMP6s*) and cytosol (*NBT:DsRed*) labeling (N=6 larvae). c) Uniform spheroid disappearance (white arrowhead) observed in both the neuronal calcium (*HuC:GCaMP6s*) and cytosol (*NBT:DsRed*) channels (N=6 larvae).
