# Supplementary material for: Axonal spheroids are regulated by Schwann cells after peripheral nerve injury
Source: bioRxiv. 2024 Nov 8:2024.11.08.622649. Preprint. [Version 1] doi: 10.1101/2024.11.08.622649 (PMC11581001; doi:10.1101/2024.11.08.622649)
Supplement: Supplement 13 [file media-13.pdf]

Figure 1: Axonal spheroids are present before and after total nerve perforation. a) A model to study spheroids *in vivo*: the posterior lateral line nerve (pLLN) of 3dpf zebrafish is transected using laser axotomy, then imaged with time-lapse, confocal microscopy centered 200 µm distal to injury site. b) Axons (*NBT:DsRed*) give rise to calcium-enriched axonal spheroids (*HuC:GCamp6s*, white arrowheads, N=6 fish). c) Calcium transients appearing in pLLN axons after injury (h:mm), followed by axon perforation (solid vs. dashed lines). d) Spheroids as a percentage of maximum quantity over time after injury (N=10 injuries, 2 no injury control fish, error bars = SEM) and times at which the last visible axon is perforated, total nerve perforation (N=15 fish, error bars = SEM). e) Histogram of spheroid lifetimes (minutes, N=92 spheroids from 7 fish).

Figure 2: Shrinkage, breakdown, or uniform disappearance defines spheroid fates. a) GCaMP6s-labeled spheroid shrinking (arrowhead) a') Kymograph showing shrinking spheroid indicated in a. a'') The ROI in a and an orthogonal view of the ROI in a showing the shrinking spheroid (magenta arrowhead) remains within the z planes captured. b) GCaMP6s-labeled spheroids break down into smaller puncta (arrowheads). c) GCaMP6s-labeled spheroids undergo uniform disappearance, or signal loss with no apparent morphological changes (arrowheads). d) Frequency of spheroid fates plotted by larva and e) averaged together (N=148 spheroids from 5 fish).

Figure 3: The extra-axonal environment is required for spheroid shrinking and breakdown. a) A model to study axonal spheroids *in vitro*: sympathetic neurons are plated in one chamber of a microfluidic device (MFD), then given 5-7 days for axons to grow across grooves into the opposite chamber. Cell bodies are aspirated, leaving behind the injured axons. Axons are imaged with time-lapse confocal microscopy using additional fluorescent dyes. b) Representative image of calcium-labeled (Fluo-4 AM) spheroids (arrowheads) with negative dextran labeling after injury *in vitro*. c) Fluo-4 AM-labeled spheroid (arrowheads) shrinking *in vitro*. d) A Fluo-4 AM-labeled spheroid undergoing uniform disappearance (arrowheads) *in vitro*. e) The frequencies of spheroid shrinking or breakdown in vitro and in vivo (N=3 MFDs and 6 larvae, p=0.0238, two-tailed Mann-Whitney U test). f) The frequencies of spheroid uniform disappearance in vitro and in vivo (N=3 MFDs and 6 larvae, p=0.0238, two-tailed Mann-Whitney U test). g) Relative frequencies of shrinking/breakdown and uniform disappearance *in vitro* and *in vivo* (N=3 MFDs and 6 larvae, p<0.0001, two-sided Fisher's Exact Test).

Figure 4: Schwann cells interact with axonal spheroids. a) Spheroids (HuC*:GCamp6s*) localize within Schwann cells (*sox10:mRFP*, white arrowheads. Maximum z projection, top, and single z plane, bottom). b) 3D projection of a) showing spheroids (black arrowheads) within Schwann cell membranes. c) Percentages of spheroid surfaces covered by Schwann cell, or overlap ratio (N=1,067 spheroids from 6 fish, error bar = SEM). d) Histogram of individual spheroid overlap ratios in c). e) A spheroid (*HuC:GCamp6s*, white arrowhead) is brought into the Schwann cell cytoplasm (*sox10:Gal4;UAS:NTR-mCherry*). f) Single plane XY (square panel), YZ (magenta), and XZ (orange) cross sections of the spheroid in e inside Schwann cell cytoplasm.

Figure 5: Spheroids expose phosphatidylserine. a) Axonal spheroids expose phosphatidylserine (PS, annexin V, white arrowheads) after *in vitro* axotomy, dashed lines indicate negative axon staining (dextran, N=3 MFDs). b) Spheroids (*HuC:GCaMP6s*) expose phosphatidylserine after axotomy *in vivo* (*s1101:Gal4;UAS:SecA5-YFP*, white arrowheads, inset showing single z slice, N=5 larvae). c) Inset from b. d) 3D rendering of b showing PS-exposing spheroids (black arrowheads).

Figure 6: Schwann cells induce shrinking and breakdown in spheroids. a) pLLNs (*HuC:GCamp6s*) from larvae treated 2-3dpf with 4mM Ronidazole (RDZ) or vehicle control (VC) to ablate Schwann cells (SCs, *sox10:Gal4; UAS:NTR-mCherry*). Spheroids frequently undergo shrinking (pink arrowheads) and breakdown (pink to white and magenta arrowheads) in VC treatment, and uniform disappearance (orange arrowheads) in RDZ treatment. b) Spheroid quantities over time (thin lines) predict significantly different values (thick lines) between treatment groups (N=14 RDZ, 6 VC, $p<0.005$, Poisson mixed-effects model, Supplementary Materials 2). c) Average spheroid lifetimes (N=7 VC, 8 RDZ larvae, $p=0.0033$, two-tailed Mann-Whitney U Test). d) Frequences of spheroid shrinking or breakdown (N=9 VC, 13 RDZ, p=0.0335, two-tailed Mann-Whitney U Test). e) Relative frequencies of shrinking/breakdown and uniform disappearance (N=9 VC, 13 RDZ, p=0.0044, two-sided Fisher's Exact Test).

Figure 7: Working model for Schwann cell effects on spheroid fates. Following injury, spheroids are more likely to undergo shrinking and breakdown when Schwann cells (SCs) are present on degenerating axons. When Schwann cells are absent, spheroids are more likely to undergo uniform disappearance. Spheroids expose phosphatidylserine, which could facilitate interactions between spheroids and SCs.

Supplementary Figure 1: GCaMP6s is a valid marker of nerve perforation. a) Intact (solid lines) and perforated (dashed lines) axons (arrowheads) detected by *NBT:DsRed* (left column) and by *HuC:GCaMP6s* (middle column) do not significantly differ in time to nerve perforation (b, N=7 larvae, $p=0.6250$, two-tailed Wilcoxon matched-pairs signed rank test).

Supplementary Figure 2: Automated spheroid detection predicts manually detected spheroid quantity. a) Automated spheroid detection precision averaged from each larva (N=8 larvae). A blinded reviewer manually identified spheroids in algorithm training videos at selected time points: a frame with maximal spheroid signal intensity and morphological variety between 15 and 90 minutes post-injury, at 150 minutes post-injury, and at 300 minutes post-injury. Spheroids detected by the algorithm were considered true positives (*TP*), those detected only by the automated method were false positives (*FP*), and those detected only by manual method were false negatives (*FN*). Precision was calculated as $TP / (TP + FP)$. b) Precision at each time after injury (N=8 larvae, p=0.2354, Friedman test). c) Automated spheroid detection sensitivity from each larva (N=8 larvae). Sensitivity was calculated as $TP / (TP+FN)$. d) Sensitivity at each time after injury (N=8 larvae, p=0.2823, one-way ANOVA). e) Manual versus automated spheroid counts at <1:30, 2:30, and 5:00 hours post-injury (points) fit with a linear mixed effect model to take into account repeated measurements from each fish (line, Supplementary Materials 1). f)

A normal Q-Q plot showing residuals from the observed values to the predicted values in e. g) A summary of the linear mixed effect model in g. For every manually detected spheroid, 0.34389 spheroids were automatically detected (N=8 larvae, 3 time points from each larva, *p*=0.0012, Satterthwaite's method)

Supplementary Figure 3: Spheroid fates are visible with both *HuC:GCaMP6s* and *NBT:DsRed* labeling. a) Spheroids shrinking (white arrowsheads) in both the neuronal calcium (*HuC:GCaMP6s*) and cytosol (*NBT:DsRed*) channels (N=6 larvae). b) A spheroid (pink arrowhead) fragments (magenta and white arrowheads) visibly with both neuronal calcium (*HuC:GCaMP6s*) and cytosol (*NBT:DsRed*) labeling (N=6 larvae). c) Uniform spheroid disappearance (white arrowhead) observed in both the neuronal calcium (*HuC:GCaMP6s*) and cytosol (*NBT:DsRed*) channels (N=6 larvae).

Supplementary video 1: Spheroids shrink. A *HuC:GCaMP6s*-labeled spheroid (arrowhead) shrinks after pLLN axotomy.

Supplementary video 2: Spheroids break down. A *HuC:GCaMP6s*-labeled spheroid (arrowhead) undergoes a series of morphological changes, breaking down from one uniform spherical shape into multiple smaller bodies.

Supplementary video 3: Spheroids undergo uniform disappearance. A *HuC:GCaMP6s*-labeled spheroid (arrowhead) disappears uniformly, or without any apparent morphological change.

Supplementary video 4: Schwann cell interactions with spheroids occur intracellularly. *HuC:GCamp6s*- (cyan) labeled spheroids (arrowheads) move off of their axons and inside of Schwann cell cytoplasm (*sox10:Gal4;UAS:NTR-mCherry,* yellow).

Supplementary video 5: Spheroids expose phosphatidylserine *in vivo.* Phosphatidylserine-labeled spheroids appear in a beads-on-a-string pattern (arrowheads in phosphatidylserine channel). *HuC:jRGECO1b* (cyan)- labeled spheroids are surrounded by phosphatidylserine (*Et(s1101:Gal4);UAS:SecA5-YFP*, yellow, arrowheads in merged channel).

Supplementary video 6: Spheroids persist in the presence of Schwann cells. *HuC:GCaMP6s*-labeled spheroids persist as smaller, broken down spheroids out to later times after pLLN injury in a vehicle control-treated (*sox10:Gal4;UAS:NTR*-mCherry) larva.

Supplementary video 7: Spheroids do not persist as long and undergo uniform disappearance more in the absence of Schwann cells. *HuC:GCaMP6s*-labeled spheroids disappear more uniformly and more rapidly after pLLN injury in a Ronidazole-treated (*sox10:Gal4;UAS:NTR*-mCherry) larva.
